# Lauric acid in crown daisy root exudate potently regulates root-knot nematode chemotaxis and disrupts *Mi-flp-18* expression to block infection

**DOI:** 10.1093/jxb/ert356

**Published:** 2013-10-29

**Authors:** Linlin Dong, Xiaolin Li, Li Huang, Ying Gao, Lina Zhong, Yuanyuan Zheng, Yuanmei Zuo

**Affiliations:** Key Laboratory of Plant–Soil Interactions, Ministry of Education, Center for Resources, Environment and Food Security, China Agricultural University, Beijing 100193, China

**Keywords:** Chemotaxis, crown daisy, lauric acid, *Meloidogyne incognita*, *Mi-flp-18*, root exudate.

## Abstract

Tomato (*Solanum lycopersicum*) crops can be severely damaged due to parasitism by the root-knot nematode (RKN) *Meloidogyne incognita*, but are protected when intercropped with crown daisy (*Chrysanthemum coronarium* L.). Root exudate may be the determining factor for this protection. An experiment using pots linked by a tube and Petri dish experiments were undertaken to confirm that tomato–crown daisy intercropping root exudate decreased the number of nematodes and alleviated nematode damage, and to determine crown daisy root exudate-regulated nematode chemotaxis. Following a gas chromatography–mass spectrometry assay, it was found that the intercropping protection was derived from the potent bioactivity of a specific root exudate component of crown daisy, namely lauric acid. The *Mi-flp-18* gene, encoding an FMRFamide-like peptide neuromodulator, regulated nematode chemotaxis and infection by RNA interference. Moreover, it was shown that lauric acid acts as both a lethal trap and a repellent for *M*. *incognita* by specifically regulating *Mi-flp-18* expression in a concentration-dependent manner. Low concentrations of lauric acid (0.5–2.0mM) attract *M*. *incognita* and consequently cause death, while high concentrations (4.0mM) repel *M*. *incognita*. This study elucidates how lauric acid in crown daisy root exudate regulates nematode chemotaxis and disrupts *Mi-flp-18* expression to alleviate nematode damage, and presents a general methodology for studying signalling systems affected by plant root exudates in the rhizosphere. This could lead to the development of economical and feasible strategies for controlling plant-parasitic nematodes, and provide an alternative to the use of pesticides in farming systems.

## Abstract (precis)

Lauric acid is a bioactive root exudate component in crown daisy. *Mi-flp-18* is a pivotal gene regualting nematode chemotaxis and infection. Lauric acid regulates the nematode chemotaxis and disrupts the *Mi-flp-18* expression in a concentration-dependent manner

## Introduction

Endoparasitic root-knot nematodes (RKNs, *Meloidogyne* species) have broad host plant specificity and are responsible for >US$125 billion annually in world-wide crop losses (Chitwood, 2003). The most damaging of all plant-parasitic nematodes is the southern RKN *M*. *incognita*, which infects almost all agricultural plants (Abad *et al.*, 2008). Tomato is one of the most popular vegetables throughout the world. However, tomato is highly susceptible to RKN infestation, which reduces crop yields and results in significant economic losses (Bird and Kaloshian, 2003). Although chemical nematicides effectively control parasitic nematodes, they are being withdrawn due to their human and environmental toxicity (Abad *et al.*, 2008). It is therefore important to identify safe and effective control strategies that have low toxicity to staple crops, humans, and animals. The intercropping of tomato–crown daisy (a popular and sustainable cropping system in Chinese greenhouse environments) reduces nematode infection and maintains profitability for farmers, because the crown daisy itself is a popular vegetable and cash crop in China (Tian *et al.*, 2011; Dong *et al.*, 2012). Crown daisy crops can be used as a biological alternative to commercial pesticides to reduce *M*. *incognita* infection, and create a ‘natural’ pest–predator relationship. However, the mechanism involved in this process remains unclear. When using the intercropping technique to control parasitic nematodes, it has been suggested that root exudate may play an important role in plant–nematode interactions in the rhizosphere (Chitwood, 2002). Specific root exudates such as tannic acid, flavonoids, glycosides, and fatty acids may regulate parasitic second-stage juvenile (J2) nematode chemotaxis by repulsion or attraction (Chitwood, 2002; Bais *et al.*, 2006). It is hypothesized that a series of bioactive compounds from crown daisy root exudate have a crucial role in suppressing parasitic nematodes in the rhizosphere. To the best of the authors’ knowledge, a specific crown daisy root exudate that decreases nematode damage has not been identified, and the effects of specific bioactive components on parasitic nematodes have also not been determined.

Infective second-stage juvenile nematodes (J2s) hatch in soil, recognize the signals emanating from host plants using a complex array of chemosensory neurons, and establish a permanent feeding site (Bird, 2004; Cortese *et al.*, 2006). Mediating the J2 chemotaxis and inhibiting the J2 infection to the host are effective measures to reduce damage by RKNs. Therein, FMRFamide-like peptides (FLPs) are a large family of small, secreted neuromodulator molecules expressed in nematodes that have diverse physiological effects on locomotion, feeding, and reproductive musculature (Johnston *et al.*, 2010). These neuropeptide signalling systems can be used as a target for anthelmintic drugs (Kimber *et al.*, 2007). A number of studies in the model species *Caenorhabditis elegans* have identified at least 29 *flp* genes, encoding >68 peptides (Li *et al.*, 1999; McVeigh *et al.*, 2006). In *C*. *elegans*, localization studies have indicated that the *flp-18* gene is involved in chemotaxis, movement, and feeding (Rogers *et al.*, 2003). In addition, *flp-18* encodes six putative peptides that activate G-protein-coupled receptors and contains a C-terminal conserved PGVLRF motif (Li *et al.*, 1999). A previous analysis of the *M*. *incognita* genome revealed that it possesses a smaller complement of 19 *flp* genes (Abad *et al.*, 2008), and that *Mi*-*flp-18* itself (GenBank accession no. AY729022) encodes five FLPs that share four C-terminal PGVLRFa motifs (McVeigh *et al.*, 2005). Recently, there has been an increase in the information available relating to the neurons, circuits, and transmitters involved in the sensory systems of *C*. *elegans* (Macosko *et al.*, 2009; Holden-Dye and Walker, 2011). However, there is little functional information relating to *flp-18* in *M*. *incognita*, and, to date, no direct evidence of *M*. *incognita flp-18* regulation by active compounds has been identified. Determining the function of *Mi-flp-18* in the interaction between nematodes and active compounds would facilitate the development of new strategies for managing parasitic nematodes.

In this study, investigations were carried out to determine how crown daisy root exudate disrupted *M*. *incognita* chemotaxis and decreased nematode infection in controlled and soil conditions, to identify and quantify compounds exuded from crown daisy roots, to determine the function of the *Mi-flp-18* gene in chemotaxis and infection, and to confirm the compounds that influenced nematode chemotaxis and interfered with *Mi-flp-18* expression to disrupt nematode infection. The results improved understanding of the molecular biology and physiology of *M*. *incognita*, and provided important principles for studies of the interactions between plants and nematodes. This could lead to the development of economical and feasible strategies for controlling plant-parasitic nematodes, and provide an alternative to the use of pesticides in farming systems.

## Materials and methods

### Nematodes and plant species

For details regarding the cultivation of *M*. *incognita* and the treatment of tomato and crown daisy seeds, refer to the description by Dong *et al.* (2012).

### Analyses of root exudate regulating nematode behaviour

**Fig. 1.**
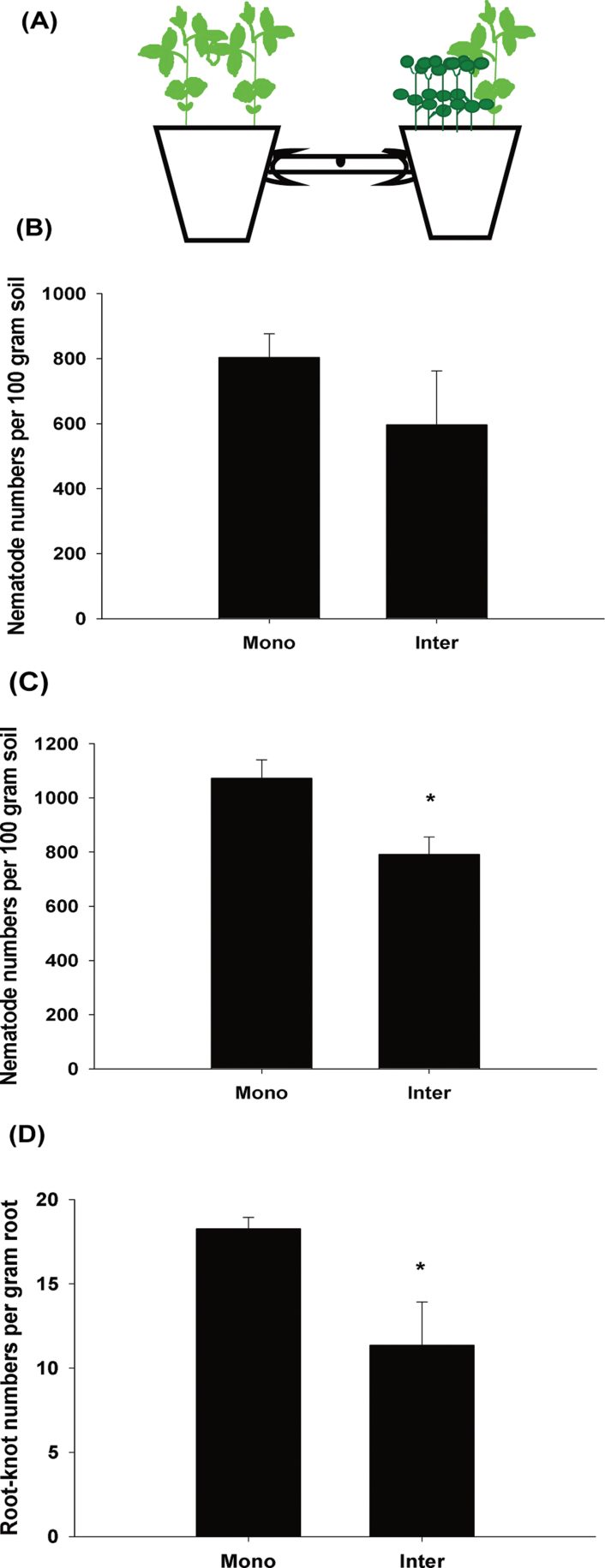
Root exudate reduced *M*. *incognita* numbers and suppressed nematode infection. Mono, monocropping; Inter, intercropping. (A) Diagram of the pot experiment linked by a tube, with the monocropping system in the left pot and the intercropping system in the right pot. (B, C) Root exudate reduced the number of nematodes in the tube and soils in the pot 35 d after inoculation. (D) Root exudate suppressed nematode infection by reducing root-knot numbers. The value of each bar represents the mean ±SE of *n*=4, where an asterisk denotes a significant difference at *P* < 0.05.

The pot experiment was performed under greenhouse conditions, as described by Dong *et al.* (2012). The design of the pot experiment linked by a tube is displayed in Fig. 1A, and in Supplementary Fig. S1A available at *JXB* online. A PVC tube (20cm length and 3cm diameter) connected two plastic pots (16cm diameter). There was a 1cm diameter hole in the middle of the PVC tube. The tube was covered at both ends with a 7mm mesh plastic net. Irradiated soil (1kg) was placed in each pot and the lower half of the PVC tube was also filled with irradiated soil. Tomato seedlings grown for 30 d were transplanted as follows: two into the left pot (monocropping condition) and one into the right pot, in which five crown daisy seeds were also sown (intercropping condition). In total, 2000 J2s were injected into the hole in the middle of the PVC tube 3 weeks after transplantation. In one combination, two tomato seedlings were planted in the left or right pot, respectively (T/T–T/T), to verify whether the presence of the same plants affected J2 numbers and infection. In another combination, two tomato seedlings were cultivated in the left pot and one tomato seedling was planted in the right pot (T/T–T). The T/T–T combination was used to confirm whether different host numbers produced the same or similar effects on the nematode numbers and infection in the pot experiment.

Tomato RKNs were collected 35 d after inoculation, and their numbers were calculated. Total RNA was extracted from tomato RKNs with TRIzol reagent (Invitrogen, Carlsbad, CA, USA), purified by RNase-free DNase I (Takara, Kyoto, Japan), and reverse transcribed by M-MLV (Promega, Fitchburg, WI, USA) to analyse *Mi-flp-18* gene expression. Soil samples were collected from the PVC tubes and pots. The PVC tubes were cut from the hole to collect the soil in both sides. Nematode populations were extracted as described by Hooper (1984). Each treatment had four replications.

Petri dish experiments were performed to confirm the direct effects of root exudate in plants on the chemotaxis of *M. incognita*. When all sterilized seeds were germinated, tomato (one plant) and crown daisy (one plant) were placed on the surface of Murashige and Skoog (MS) medium to determine the level of *M. incognita* chemotaxis by Petri dish (9cm diameter) experiment. After 5 d, 50 fresh *M*. *incognita* J2s were released onto the MS medium 1cm from the root tips of different seedlings. After 4h, nematode numbers within the area 0.5cm from the roots were observed using a Leica Z16 microscope and counted. The number of nematodes within 0.5cm of the root divided by the total number of nematodes was used as an attractiveness index. The data were the mean of four independent experiments.

### Collection and screening of root exudate

Root exudate was collected as described by Tang and Young (1982), with minor modifications. Briefly, tomato seedlings (five plants) and crown daisy (15 plants) grown for 30 d were transplanted into pots (2 litres) of hydroponic culture in full-strength nutrient solution (Pramanik *et al.*, 2000) with a pH of 6.8. Daily loss was compensated with distilled water and full-strength nutrient concentrations to maintain steady nutrient concentrations and pH during cultivation. After being placed into the hydroponic culture for 30 d, the nutrient solutions were passed through columns packed with 20cm^3^ Amberlite XAD-4 resin (Sigma-Aldrich) as an adsorbent. The used resins were washed in 800ml of methyl alcohol and eluates were dried by rotary evaporation *in vacuo* at 40 °C. Residues were dissolved in 1ml of methyl alcohol and loaded on an SPE silica gel column (Agilent, Santa Clara, CA, USA). The column was washed separately with 6ml of solvents with various polarities in the following order: chloroform, ethyl acetate, acetone, and methyl alcohol. Trapping systems lacking plant species cultures were used as controls. Each treatment had three replications.

All root exudate samples were analysed using gas chromatography–mass spectrometry (GC-MS; Agilent, HP-5ms, equipped with a 0.25 mm×30 m×0.25 μm capillary column) with helium as the carrier gas. The initial oven temperature was maintained at 60 °C for 1min by cryogenic cooling. The oven temperature was increased to 200 °C at a rate of 8 °C min^–1^, maintained for 2min, and then increased to a final temperature of 280 °C at a rate of 15°C min^–1^, which was maintained for 10min. The injection port temperature was set at 250 °C. The helium carrier gas linear velocity was kept at 1.0ml min^–1^ by automated pressure control. Detection was achieved by mass selective detection in scan mode. All exudate collection solutions were identified by searching the mass spectral database and quantified based on their GC-MS response compared with the mass spectra and the retention time of a standard of known concentration.

### Analysis of *Mi-flp-18* function

Total RNA was extracted from 10000 J2s using an RNease kit (Tiangen, China). Following reverse transcription, *M. incognita* cDNA was used as a template to synthesize double-stranded RNA (dsRNA) with primers targeting the *Mi*-*flp-18* gene sequence (*flp-18*-F and *flp-18*-R listed in Supplementary Table S1 at *JXB* online) to amplify a 483bp target fragment. *Mi*-*flp-18* dsRNA was synthesized from PCR products amplified with the T7 promoter sequence incorporated at the 5′ end (T7 *flp-18*-F and T7 *flp-18*-R listed in Supplementary Table S1). PCR was performed using 5U of *Taq* DNA polymerase (Genestar). The PCR products were analysed by running a 2 μl aliquot in a 1% agarose gel, and the remainder were purified using a PCR purification kit (Tiangen). PCR products (2 μg) were used as templates to synthesize *Mi-flp-18* RNAs according to the MEGAsript RNAi kit protocol (Ambion, Austin, TX, USA). The *gfp* gene was cloned from the binary vector pCAMBIA (Takara, Japan), and the primers used are listed in Supplementary Table S1. Products of *gfp* (green fluorescent protein) dsRNA served as a control, and the synthesis of *gfp* dsRNA followed the process described above.

Fluorescein isothiocyanate (FITC) was used as a tracer to assess uptake efficiency. Freshly hatched J2s were immersed in 1mg ml^–1^ *Mi-flp-18* dsRNA in a soaking buffer with FITC (0.1mg ml^–1^), 0.5% resorcinol, and 30mM octopamine. Soaked J2s were incubated for 6h in the dark at room temperature on a rotator. As controls, J2s were incubated in soaking buffer alone or in soaking buffer with *gfp* dsRNA. After incubation, J2s were washed five times with nuclease-free water by centrifugation. Treated nematodes were observed using an Olympus BX51 fluorescence microscope to determine uptake efficiency.

Total RNA was extracted from 10000 J2s that were soaked for 6h in soaking buffer with 1mg ml^–1^ *gfp* dsRNA, soaking buffer alone, or soaking buffer with 1mg ml^–1^ *Mi-flp-18* dsRNA. The converted first-strand cDNA was used as a template for real-time PCR assay. The primers (re-*flp-18*-F, re-*flp-18*-R listed in Supplementary Table S1 at *JXB* online) were designed for the 5′ end of *Mi-flp-18*, outside of the region used for dsRNA synthesis. The relative expression of other *Mi-flp* genes in the treated nematodes was also determined by real-time PCR assay (primers listed in Supplementary Table S1). The data were the mean of three independent experiments.

To examine the effects of RNA interference (RNAi) on nematode chemotaxis, 50 J2s from each of three soaking treatments (soaking buffer with *gfp* dsRNA, soaking buffer alone, or soaking buffer with 1mg ml^–1^ *Mi-flp-18* dsRNA) were placed on MS medium (9cm diameter Petri dish) 1cm from tomato root tips to determine the attractiveness index. Differential effects of RNAi on J2 pathogenicity were calculated by taking three uniform tomato seedlings planted 30 d previously and transplanting them into one pot. Two weeks after transplanting, 2000 J2s soaked in each of the three treatments (soaking buffer with *gfp* dsRNA, soaking buffer alone, or soaking buffer with 1mg ml^–1^ *Mi-flp-18* dsRNA) were inoculated into each pot. The number of root-knots was calculated 35 d after inoculation, to determine the effects of RNAi on infection with treated J2s on tomato plants. Each treatment had three replications.

### Chemicals and chemotaxis assays

Lauric acid (Sigma-Aldrich) was diluted to 4.0mM in methyl alcohol as a stock solution, which itself was diluted in methyl alcohol to 0.5, 1.0, and 2.0mM, and stored at –20 °C.

Chemotaxis assays using Petri dishes were conducted according to previous reports (Ward, 1973; Bargmann *et al.*, 1993; O’Halloran and Burnell, 2003), with some modifications. Briefly, 10 μl of screening chemicals were placed on the agar surface over the centre of one circle, and 10 μl of methyl alcohol (as a control) was placed on a second circle. The numbers of immobilized nematodes on the treated and control circles were determined using a stereomicroscope. The chemotaxis index was calculated following Bargmann and Horvitz (1991). It was found that lauric acid was lethal for J2s, and the death rate was calculated as the number of dead nematodes in a circle containing lauric acid divided by the total number of nematodes in that circle. In addition, 50 μl of each substance (0, 0.5, 1.0, 2.0, and 4.0mM) were added, respectively, to both circles in one plate, and 1000 J2s were incubated in the centre of the plate as in the process described above, and later collected for gene expression analysis. All data were the mean of four independent experiments.

### SYBR Green real-time PCR experiments

Real-time PCR was conducted using an iQ™5 Multicolor Real-time PCR Detection System (Bio-Rad, Hercules, CA, USA). Each reaction was performed using a SYBR Green PCR Master mix (Toyobo, Osaka, Japan). A region of the *M*. *incognita actin* gene (GenBank accession no. BE225475) was used as a control. The transcript abundance was normalized to nematode *actin* (Luo *et al.*, 2005) at the transcript level.

### Statistical analysis

SPSS 11.0 was used for statistical analyses. The parameters were calculated for all replicates of treatments and subjected to an analysis of variance (ANOVA). Mean values were compared by calculating the least significant difference (LSD) at the 5% level or reported as significant or non-significant by paired *t*-tests (*P* < 0.05).

## Results

### Root exudate regulates *M*. *incognita* chemotaxis and reduces the tomato root-knots

The relevant physiological mechanisms underlying the effects of crown daisy on *M*. *incognita* were examined by using a novel and specific experiment using pots linked by a tube (Fig. 1A; Supplementary Fig. S1A at *JXB* online). There were 25.8% fewer nematodes in the tube near the tomato–crown daisy intercropping system than near the monoculture system 35 d after inoculation (Fig. 1B); 26.2% fewer nematodes inside the intercropping pot than inside the monoculture pot (Fig. 1C); and 37.8% fewer tomato root-knots in the intercropping system than in the monoculture system (Fig. 1D). As a control, two tomato plants were evaluated versus tomato plants (two or one) and it was found that the variation in nematode and root-knot numbers between the two sides was not significant (Supplementary Fig. S1B–D). These results suggest that the root exudate of the combination (T/T–T or T/T–T/T) had no significant impact on the number of nematodes and infection in the pot experiment, and the root exudate in the intercropping system decreased the number of nematodes in the soil and alleviated nematode infection on the host.

To investigate directly the effects of root exudate on nematode chemotaxis, Petri dish experiments that eliminated confounding environmental factors were performed and it was confirmed that the root exudate of tomato and crown daisy regulated J2 chemotaxis (4h after inoculation, J2s were observed around tomato roots but not crown daisy roots, with a attractiveness index of 0.15 and 0, respectively; Table 1; and Supplementary Fig. S1E at *JXB* online). These results suggest that tomato root exudate attracts nematodes while crown daisy root exudate repels them.

**Table 1.** *Root exudate in tomato and crown daisy regulates chemotaxis of* M. incognita *J2s*

Plant species	Attractiveness index
Tomato	0.15±0.03
Crown daisy	0±0*

The data represent the means ±SE of *n*=4.

*Significant difference at *P* < 0.05.

### Lauric acid is a bioactive compound in crown daisy root exudate

**Fig. 2.**
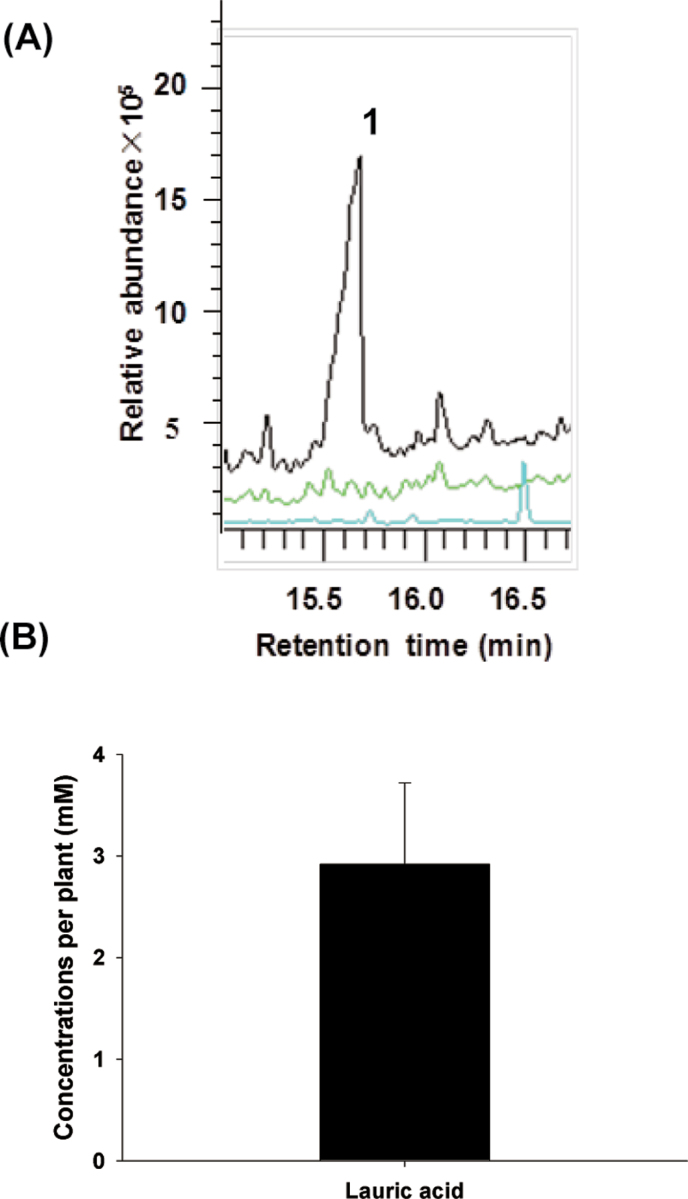
Identification and quantification of root exudate from tomato and crown daisy. (A) GC-MS chromatogram of tomato and crown daisy root exudate (black, green, and blue lines represent crown daisy, tomato, and the control, respectively). (B) Quantification of lauric acid in crown daisy root exudate. 1, lauric acid. The value of each bar represents the mean ±SE of *n*=3.

To identify the specific compounds from crown daisy that regulate nematode chemotaxis, a highly sensitive GC-MS quantification method was used to identify root exudate from crown daisy. Unique peaks were detected in chloroform from crown daisy, but not from tomato or control samples (Supplementary Fig. S3 at *JXB* online). Based on the higher relative abundance of the peaks, and by analysing the mass spectral database (matched score >95%), a peak was detected at 15.6min, which was identified as lauric acid (Fig. 2A). Comparing the mass spectra and retention time with a standard of known concentration, it was determined that up to 2.92mM lauric acid per plant was exuded and accumulated 30 d after transplantation (Fig. 2B).

### RNAi disrupts *Mi-flp-18* expression at the transcript level during the J2 stage

**Fig. 3.**
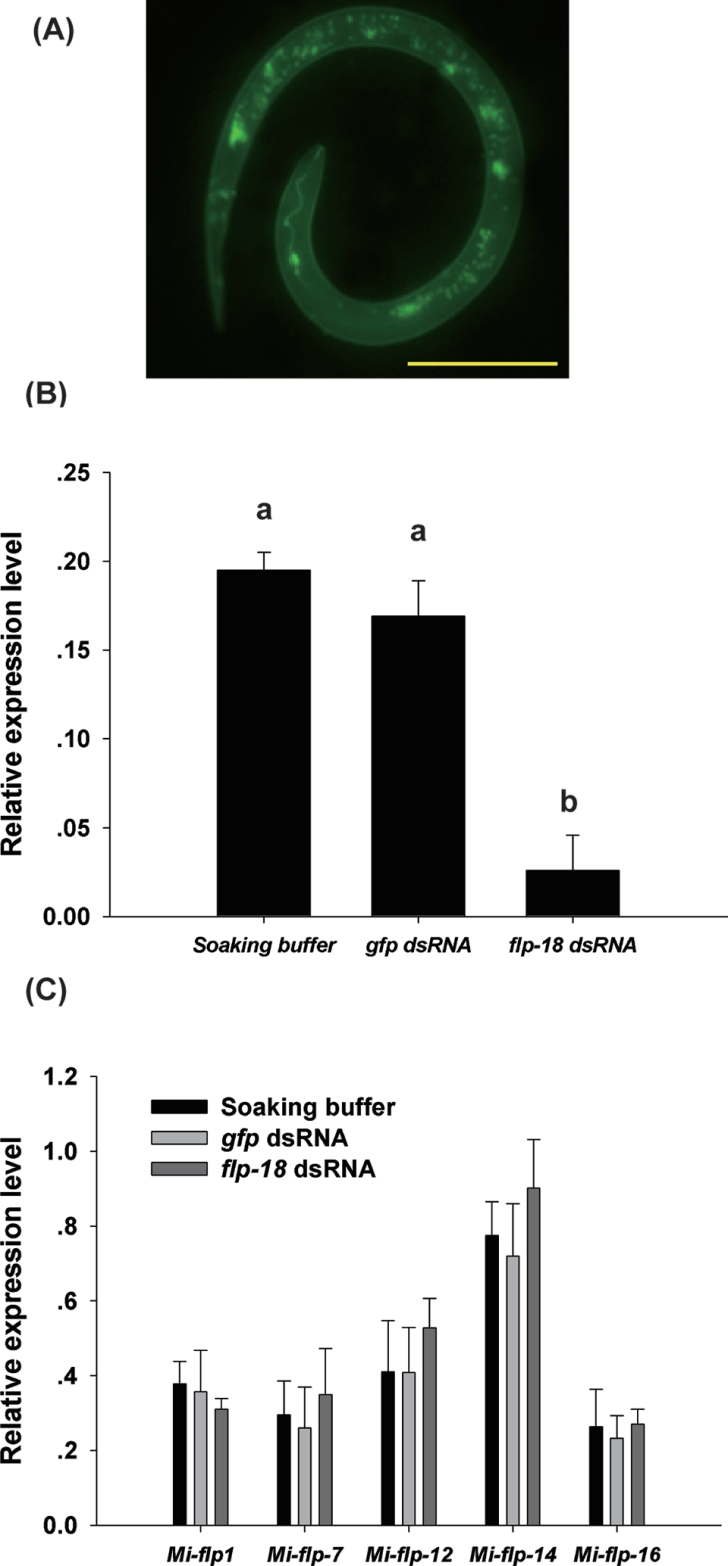
RNAi silencing of *Mi-flp-18* in *M*. *incognita* J2. Soaking buffe, treatment with soaking buffer alone; *gfp* dsRNA, treatment with soaking buffer containing 1mg ml^–1^ *gfp* dsRNA; *flp-18* dsRNA, treatment with soaking buffer containing 1mg ml^–1^ *Mi-flp-18* dsRNA. (A) Fluorescence microscopy showing the ingestion of FITC in the soaking buffer by *M*. *incognita* J2s (scale bar, 10 μm). (B) Real-time PCR analysis of *Mi-flp-18* transcript abundance. (C) Effects of *Mi-flp-18* dsRNA on *Mi-flp* gene expression. The value of each bar represents the mean ±SE of *n*=3, where bars with different letters denote a significant difference at *P* < 0.05.

RNAi was used to determine the role of the *Mi-flp-18* gene in the response of *M. incognita* J2s to root exudate from plants. Fluorescence microscopy showed that J2s efficiently absorbed dsRNA (Fig. 3A). Real-time PCR revealed that J2 ingestion of *Mi-flp-18* dsRNA resulted in a reduction of >85.5% in *Mi-flp-18* transcripts compared with J2s treated with soaking buffer alone or treated with *gfp* dsRNA. The difference in expression levels of J2s treated by soaking buffer with *gfp* dsRNA and soaking buffer alone was not significant (Fig. 3B). To evaluate the effects of RNAi further, the relative expression levels of several *flp* genes in J2s (*Mi-flp-1*, *Mi-flp-7*, *Mi-flp-12*, *Mi-flp-14*, and *Mi-flp-16*) treated with *Mi-flp-18* dsRNA were also analysed. The relative expression of *Mi-flp-1* was reduced by 17.9% compared with the controls; however, the relative expression of other *Mi-flp* genes increased by 2.7–34.2% compared with the J2s soaked in buffer alone or treated by soaking buffer with *gfp* dsRNA. None of these differences was significant (Fig. 3C). These results strongly suggest that soaking buffer alone and soaking buffer with *gfp* dsRNA do not markedly affect dsRNA phenotypes, the *Mi*-*flp* genes display no redundancy, and the *Mi-flp-18* RNAi effects are significant at the transcript level.

### RNAi targeting of *Mi-flp-18* inhibits *M*. *incognita* chemotaxis and infection

**Fig. 4.**
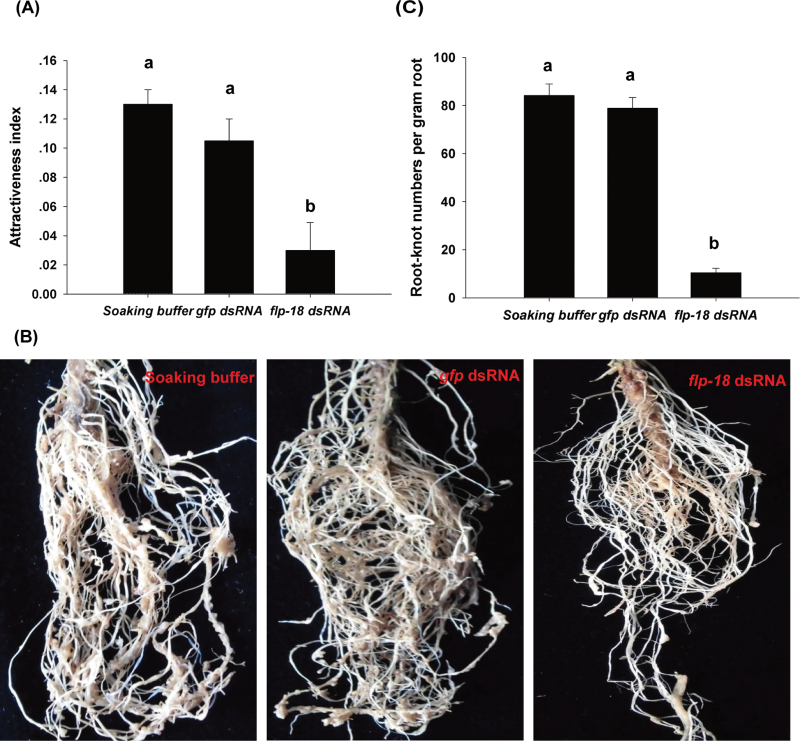
*Mi-flp-18* is a pivotal gene regulating *M. incognita* chemotaxis and infection. Soaking buffer, treatment with soaking buffer alone; *gfp* dsRNA, treatment with soaking buffer containing 1mg ml^–1^ *gfp* dsRNA; *flp-18* dsRNA, treatment with soaking buffer containing 1mg ml^–1^ *Mi-flp-18* dsRNA. (A) *Mi-flp-18* RNAi inhibited J2 chemotaxis in a Petri dish experiment. J2s immersed in three alternative treatments (soaking buffer alone, soaking buffer containing 1mg ml^–1^ *gfp* dsRNA, or soaking buffer containing 1mg ml^–1^ *Mi-flp-18* dsRNA) were transferred to the Petri dish. (B, C) In the pot experiment, J2s inoculated with *Mi-flp-18* dsRNA displayed inhibited infection ability (fewer root knot numbers). The value of each bar represents the mean ±SE of *n*=3, where bars with different letters denote a significant difference at *P* < 0.05.

*Mi-flp-18* dsRNA-treated nematodes were placed on a Petri dish 1cm from the tomato root tips to determine the effects of *Mi-flp-18* RNAi on J2 chemotaxis. Within 0.5cm of the tomato roots, the number of nematodes decreased after treatment with *Mi-flp-18* dsRNA compared with treatment with soaking buffer alone or with nematodes soaked in buffer with *gfp* dsRNA. The attractiveness index of the J2s decreased by 77.0% compared with treatment with soaking buffer alone, and a 71.4% reduction of the attractiveness index was observed when compared with the treatment with soaking buffer with *gfp* dsRNA (Fig. 4A).

In further tests, RNAi-treated nematodes were inoculated into pots to determine the effects of RNAi on nematode infection. Interestingly, 35 d after inoculation with J2s that had ingested *Mi-flp-18* dsRNA, it was observed that root-knot formation was reduced (and the knots were smaller) compared with controls (Fig. 4B). Significant reductions (86.8% and 87.4%) were found in the number of root-knots produced by *Mi-flp-18* dsRNA-treated J2s compared with those soaked in buffer with *gfp* dsRNA or buffer alone after 35 d (Fig. 4C).

The attractiveness index and root-knot production did not differ significantly between J2s treated by soaking buffer with *gfp* dsRNA and soaking buffer alone (Fig. 4). Taken together with the results above, this suggests that *Mi-flp-18* RNAi results in defective nematode chemotaxis and affects the parasitic stages. Importantly, the phenotypes of nematodes treated with RNAi suggest that *Mi-flp-18* is a novel target for parasitic nematode control.

### Lauric acid mediates nematode chemotaxis by affecting *Mi-flp-18* expression

**Fig. 5.**
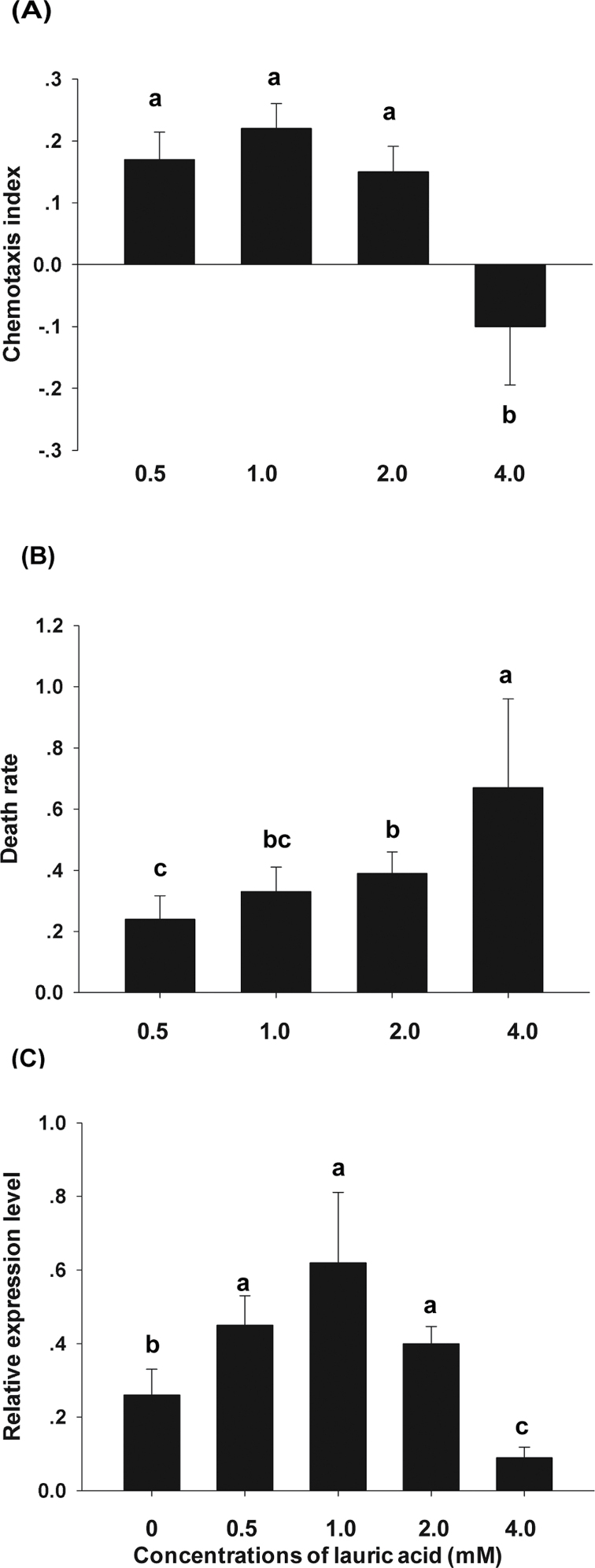
Lauric acid affected *M. incognita* chemotaxis by regulating *Mi-flp-18* expression and resulted in death. (A) Lauric acid concentrations of 0.5–4.0mM mediated J2 chemotaxis. (B) The death rate of J2s, caused by lauric acid. (C) Lauric acid regulated *Mi-flp-18* expression in J2s. The value of each bar represents the mean ±SE of *n*=4, where bars with different letters denote a significant difference at *P* < 0.05.

To determine how lauric acid affected J2 chemotaxis and regulated *Mi-flp-18* expression, the effects of lauric acid on J2 behaviour were observed 3h after inoculation by a chemotaxis assay. Lauric acid attracted or repelled J2s in a concentration-dependent manner, and subsequently had lethal results (Fig. 5). At concentrations of 0.5–2.0mM, lauric acid attracted *M*. *incognita* with an increase in the chemotaxis index from 0.17 to 0.22, while 4.0mM lauric acid significantly repelled J2s (chemotaxis index of –0.08) (Fig. 5A). Moreover, although 4.0mM lauric acid repelled the J2s, a higher nematode death rate was observed in the circles containing 4.0mM lauric acid. The death rate increased from 27.0% to 67.0% when lauric acid concentrations were increased from 0.5mM to 4.0mM, respectively (Fig. 5B). These results indicate that the preference for attraction or avoidance of J2s depends on the concentration of lauric acid. To determine how lauric acid regulates the concentration-dependent behaviour of J2s, *Mi-flp-18* expression, which has a role in J2 chemotaxis and infection, was examined (Fig. 4). It was found that *Mi-flp-18* expression was up-regulated in J2s treated with lauric acid at concentrations of 0.5–2.0mM but was reduced by 65.4% in those treated with 4.0mM lauric acid compared with the controls (Fig. 5C), which is consistent with the behaviour illustrated in Fig. 5A. These analyses indicate that lauric acid is an essential trigger that mediates nematode chemotaxis and regulates *Mi-flp-18* expression to block infection.

### Tomato–crown daisy intercropping system down-regulates *Mi-flp-18* expression in parasitic stages of *M. incognita*

**Fig. 6.**
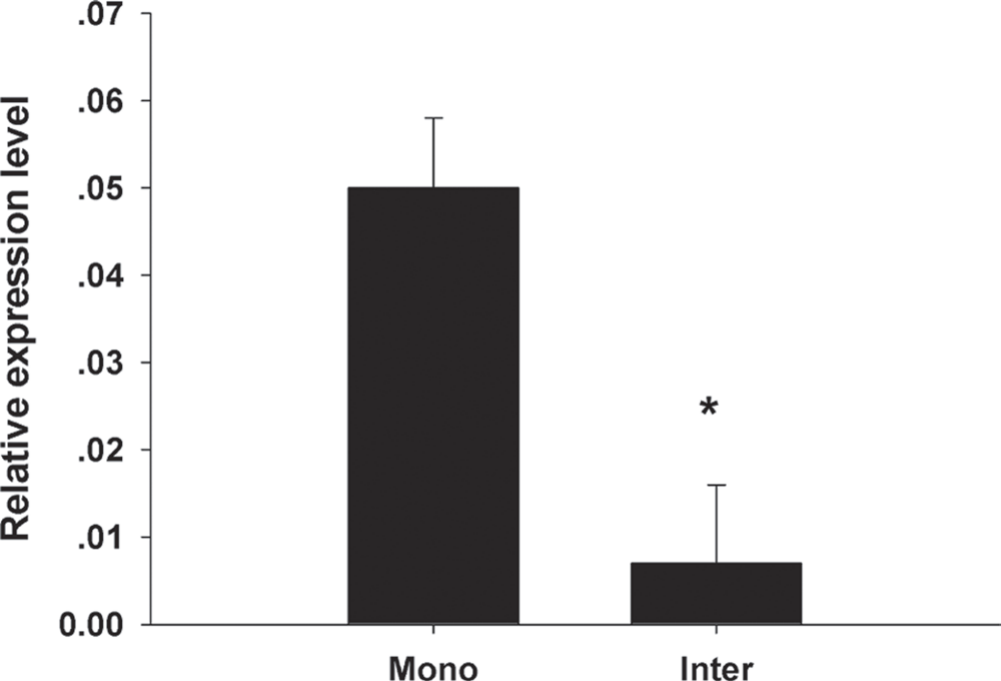
The tomato–crown daisy intercropping system inhibited *Mi-flp-18* expression in parasitism. The value of each bar represents the mean ±SE of *n*=4, where an asterisk denotes a significant difference at *P* < 0.05.

To determine the effects of the tomato–crown daisy intercropping system on the *Mi-flp-18* expression in the parasitic stages of *M. incognita* under natural soil conditions, samples of infected tomato root-knots were collected from the pot experiments and *Mi-flp-18* expression was analysed by real-time PCR assay. The tomato–crown daisy intercropping system significantly down-regulated *Mi-flp-18* expression in infected tomato roots by 86.0% at 35 d post-infection, compared with a tomato monoculture linked by a tube (Fig. 6). The *Mi-flp-18* expression in infected tomato roots from a tomato monoculture was not significant in comparison with that of a tomato monoculture linked by a tube (Supplementary Fig. S4 at *JXB* online). These results indicate that the root exudate in the intercropping system may be the factor that determines alleviation of nematode infection by inhibiting the expression of *Mi-flp-18*.

## Discussion

### Root exudate regulates *M. incognita* chemotaxis and reduces nematode infection

In the present study, it was confirmed that root exudate effectively alleviates nematode damage on the tomato plants by reducing *M. incognita* numbers and infection of the host in the intercropping system. Many studies have reported that maize intercropping with either *Canavalia ensiformis* or *Mucuna pruriens* reduces nematode populations in the roots of maize by up to 32% and nematode disease is reduced by up to 26% (Arim *et al.*, 2006; Berry *et al.*, 2009). It has been reported that intercropping may limit the food for host-specific nematodes, resulting in interspecies competition, which is considered to be the mechanism of nematode control in an intercropping system (van de Putten *et al.*, 2006). In the experiments presented here, the combination of one tomato seedling linked by two tomato seedlings displayed little difference in the number of nematodes and infection in the experiment using pots linked by a tube. The main reason for this is likely to be that the host is sufficient for the nematodes and no significant interspecies competition of nematodes was observed in the pot experiment linked by a tube. In addition, the experiment using pots linked by a tube were performed using double independent tests during 2009 and 2010. The results showed that root exudate in the tomato–crown daisy intercropping system decreased the nematode numbers, inhibited nematode infections, and down-regulated *Mi-flp-18* expression (Supplementary Fig. S2 at *JXB* online). These data indicate that the experiment using pots linked by a tube effectively reproduced the ability of root exudate to protect the host. Taken together with the previous results, this strongly suggests that the root exudate in an intercropping system decreased the number of nematodes in the soil and suppressed nematode infection of the host.

Many asteraceae have been studied for their use as co-crops to control parasitic nematodes and decrease nematode damage to the host (Bais *et al.*, 2004; Tsay *et al.*, 2004; Dong *et al.*, 2012). Crown daisy served as the intercrop and effectively decreased the nematode numbers and host infection in the pot experiment. Further Petri dish experiments directly confirmed that root exudate regulates nematode chemotaxis. Previous studies have shown that root exudate plays an important role in the communication between plants and nematodes in the rhizosphere (Hooks *et al.*, 2010). Maize root exudate emits (E)-β-caryophyllene, which regulates nematode chemotaxis (Degenhardt *et al.*, 2009). However, to the authors’ knowledge, this is the first report of an association between root exudate in an intercropping system and reductions in parasitic damage.

### Lauric acid is a special and highly abundant compound in crown daisy root exudate

It is hypothesized that discrete compounds from crown daisy root exudate may play important roles in regulating nematode behaviour, resulting in a decrease in the damage caused by nematodes to the host in a tomato–crown daisy intercropping system. A number of compounds were identified in the root exudate of tomato or crown daisy; many bioactive compounds may be determinants of alleviating nematode damage. However, only the highly abundant compound (lauric acid) which existed in the root exudate of crown daisy was screened for, relying on the mass spectral database. It is also likely that other compounds in crown daisy root exudate play important roles and, therefore, further studies are required. Although the functions of most root exudates have not been confirmed, an abundance of compounds has been detected in the root exudate (Chitwood, 2002; Horiuchi *et al.*, 2005; Curtis, 2008). Many crops naturally release nematotoxic compounds into the environment either from their roots or directly from plant tissue to suppress RKNs (Bais *et al.*, 2004; Bais *et al.*, 2006; Hooks *et al.*, 2010). It has been demonstrated that the phototoxin α-therthienyl, which has been extracted from asteraceae species, is a major nematicidal compound. This compound may be released into the environment to suppress nematode damage (Wang *et al.*, 2007). Lauric acid has been identified as a novel bioactive and high-abundance compound in root exudates of the family asteraceae. However, lauric acid was assayed in a hydroponic culture, from which environmental factors were absent, and so the accumulated lauric acid content in natural intercropping practice is unclear.

### *Mi-flp-18* is a pivotal gene regulating *M. incognita* chemotaxis and infection

To confirm the response of *M. incognita* to root exudate, the function of the *Mi-flp-18* gene was investigated by RNAi assay. Soaking nematodes in dsRNA is an effective and convenient method of evaluating gene function (Hannon, 2002; Huang *et al.*, 2006). The effects of silencing depend on the concentration and length of dsRNA, solution formulation, and the incubation time of the nematodes in dsRNA (Bakhetia *et al.*, 2005; Rosso *et al.*, 2005; Kimber *et al.*, 2007; Dalzell *et al.*, 2009). Many studies have reported significant RNAi effects at the transcript level (Huang *et al.*, 2006; Dalzell *et al.*, 2010). In the present studies, soaking buffer alone and soaking buffer with *gfp* dsRNA did not significantly affect dsRNA phenotypes and the *Mi*-*flp* genes displayed no redundancy. These results indicate that non-target dsRNA control had a lesser impact on J2s. Importantly, the defect in nematodes is phenocopied by the phenotype of *Mi-flp-18* RNAi-treated nematodes. As with the nematode *Globodera pallida*, *flp* gene knockdown by RNAi resulted in defective motor function, and *flp* genes were not redundant (Kimber *et al.*, 2007).

In the nematode *C*. *elegans*, *flp-18* is expressed in the motor neuron RIM, and the interneurons AVA, AIY, and RIG (Rogers *et al.*, 2003), and, additionally, FLPs exert a potent physiological impact on locomotion and perception (Johnston *et al.*, 2010; Holden-Dye and Walker, 2011). The *Gp-flp*-18 gene is knocked down, resulting in markedly aberrant phenotypes, which are consistent with the important role of the *Gp-flp*-18 gene in motor function (Kimber *et al.*, 2007). These results strongly suggest that *flp-18* is involved in nematode perception and movement. In this study, *Mi-flp-18* was the pivotal gene controlling J2 chemotaxis towards the host. Moreover, in *C*. *elegans*, *flp-18* is expressed in the pharyngeal neuron M2, which forms part of the pharyngeal nervous system that mediates the pumping of food into the gut (Avery and Horvitz, 1989; Rogers *et al.*, 2003). This localization suggests that FLP plays an important role in *C*. *elegans* feeding; however, the *Mi-flp-18* gene in parasitic nematodes has been shown to play a role in infection. The potential function of *Mi-flp-18* may be further elucidated by *in situ* hybridization.

### Lauric acid mediates *M. incognita* chemotaxis and regulates *Mi-flp-18* expression in a concentration-dependent manner

It was demonstrated that lauric acid, a specific crown daisy root exudate, mediates bidirectional behavioural responses of parasitic nematodes and regulates *Mi-flp-18* expression. Many studies have suggested that root exudate regulates nematode chemotaxis, and even result in death (Curtis, 2008; Lilley *et al.*, 2011). However, the molecular mechanisms of root exudate-mediated nematode chemotaxis remain unknown. Notably, the molecular mechanisms of root exudate-mediated nematode chemotaxis are absent in published reports. The *Mi-flp-18* expression of J2s treated by lauric acid increases our understanding of how root exudate regulates the host–nematode interaction at the molecular level.

In addition, previous studies have shown that an odorant can induce attractive or repulsive responses in *C*. *elegans* depending on its concentration (Troemel *et al.*, 1997; Yoshida *et al.*, 2012). The different concentrations of lauric acid used in the chemotaxis analysis may mimic the presence of root exudate in the rhizosphere. The accumulation of biologically active compounds depends on the root exudate continuously producing and secreting compounds into the rhizosphere during plant growth (Bais *et al.*, 2001; Vicre *et al.*, 2005). Hence, the density of nematode-toxic plants, and the distance between them and vulnerable plants, may play an important role in nematode control. The present results suggest that five crown daisy plants provided sufficient root exudate to block *M*. *incognita* infection of tomato in the complex soil conditions employed, and the root exudate of one crown daisy seedling was sufficient to repel nematodes in the Petri dish experiment in which environmental factors were absent. In addition, lauric acid has low toxicity to humans and the environment, and thus may represent a new and safe insecticide.

### Root exudate is presumed to regulate *M. incognita* chemotaxis and interfere with *Mi-flp-18* expression to inhibit infection in the tomato–crown daisy intercropping system

**Fig. 7.**
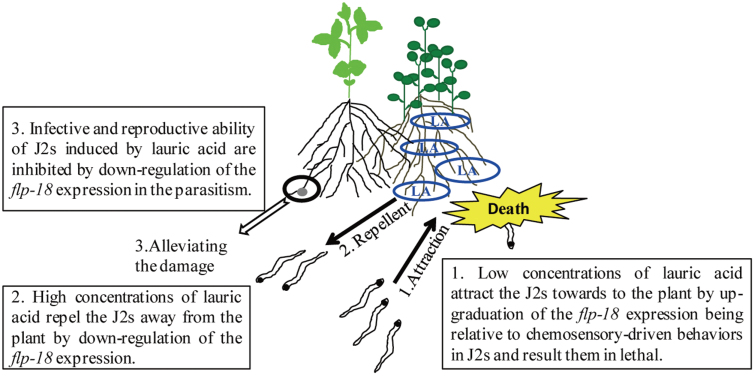
Schematic model demonstrating that root exudate in the tomato–crown daisy intercropping system may regulate J2 chemotaxis and infection by mediating *Mi-flp-18* expression. LA, lauric acid.

The tomato–crown daisy intercropping system is an ideal model for analysing the physiological and molecular mechanisms that mediate the olfactory behavioural switch in nematodes in response to root exudate. The *flp-18* expression pattern changes were dependent on the lauric acid concentration in the rhizosphere of plants. These changes probably regulate the changes in olfactory preference between lower and higher lauric acid concentrations, which in turn control nematode chemotaxis and infection (Fig. 7). The results infer that crown daisy (being a trap crop) attracts nematodes towards its roots at lower lauric acid concentrations, which are subsequently lethal to nematodes around the crown daisy. Alternatively, at higher lauric acid concentrations under natural conditions, it may act as a repellent, which results in nematodes moving away from the plant roots, reducing the nematode population near all plants in the vicinity. In addition, *Mi-flp-18* expression is down-regulated during the parasitism of J2s that encounter high concentrations of lauric acid in the tomato–crown daisy intercropping system.

In conclusion, crown daisy releases lauric acid, which regulates *M*. *incognita* chemotaxis and blocks infection, thereby reducing nematode damage. The *Mi-flp-18* gene is essential in *M*. *incognita* chemotaxis and infection, and lauric acid regulates nematode chemotaxis and interferes with *Mi-flp-18* function. These results demonstrate that crown daisy root exudate (lauric acid in particular) plays an important role in nematode chemotaxis, resulting in blocking of nematode infection. It was also found that *Mi-flp-18* is a novel target for parasitic nematode control. This study reveals new information relating to the molecular and physiological mechanisms of signal transmission between plants and nematodes in the rhizosphere. These results increase understanding of the physiology and pharmacology of nematodes, and the host–nematode interaction.

## Supplementary data

Supplementary data are available at *JXB* online.

Table S1. The primers used in this study.

Figure S1. Root exudate plays important roles in blocking *M*. *incognita* infection.

Figure S2. Root exudate in the tomato–crown daisy intercropping system reduced the number of nematodes and decreased the damage caused by nematodes by down-regulating *Mi-flp-18* expression in the pot experiment linked by a tube during 2009.

Figure S3. Gas chromatogram of root exudate from tomato and crown daisy by GC-MS assay.

Figure S4. *Mi-flp-18* expression is down-regulated during parasitism in the tomato–crown daisy intercropping system.

Supplementary Data

## References

[CIT0001] Abad P Gouzy J Aury JM 2008 Genome sequence of the metazoan plant-parasitic nematode *Meloidogyne incognita* . Nature Biotechnology 26, 909–915 10.1038/nbt.1482 18660804

[CIT0002] Arim OJ Waceke JW Waudo SW Kimenju JW 2006 Effects of *Canavalia ensiformis* and *Mucuna pruriens* intercrops on *Pratylencbus zeae* damage and yield of maize in subsistence agriculture. Plant and Soil 284, 243–251

[CIT0003] Avery L Horvitz HR 1989 Pharyngeal pumping continues after laser killing of the pharyngeal nervous system of *C*. *elegans* . Neuron 3, 473–485 2642006 10.1016/0896-6273(89)90206-7

[CIT0004] Bakhetia M Charlton WL Urwin PE McPherson MJ Atkinson HJ 2005 RNA interference and plant parasitic nematodes. Trends in Plant Science 10, 262–267 10.1016/j.tplants.2005.06.007 16027029

[CIT0005] Bais HP Loyola-Vargas VM Flores HE Vivanco JM 2001 Root-specific metabolism: the biology and biochemistry of underground organs. In Vitro Cellular and Developmental Biology-Plant 37, 730–741

[CIT0006] Bais HP Park SW Weir TL Callaway RM Vivanco JM 2004 How plants communicate using the underground information superhighway. Trends in Plant Science 9, 26–32 14729216 10.1016/j.tplants.2003.11.008

[CIT0007] Bais HP Weir TL Perry LG Gilroy S Vivanco JM 2006 The role of root exudates in rhizosphere interaction with plants and other organisms. Annual Review of Plant Biology 57, 233–266 10.1146/annurev.arplant.57.032905.105159 16669762

[CIT0008] Bargmann CI Horvitz HR 1991 Chemosensory neurons with overlapping functions direct chemotaxis to multiple chemicals in *C*. *elegans* . Neuron 7, 729–742 1660283 10.1016/0896-6273(91)90276-6

[CIT0009] Bargmann CI Hartweig E Horvitz HR 1993 Odorant selective genes and neurons mediate olfaction in *C*. *elegans* . Cell 74, 515–527 8348618 10.1016/0092-8674(93)80053-h

[CIT0010] Berry SD Dana P Spaull VW Cadet P 2009 Effect of intercropping on nematodes in two small-scale sugarcane farming systems in South Africa. Nematropica 39, 11–33

[CIT0011] Bird DM 2004 Signaling between nematodes and plants. Current Opinion in Plant Biology 7, 372–376 15231258 10.1016/j.pbi.2004.05.005

[CIT0012] Bird DM Kaloshian I 2003 Are roots special? Nematodes have their say. Physiological and Molecular Plant Pathology 62, 115–123

[CIT0013] Chitwood DJ 2002 Phytochemical-based strategies for nematode control. Annual Review of Phytopathology 40, 221–249 10.1146/annurev.phyto.40.032602.130045 12147760

[CIT0014] Chitwood DJ 2003 Research on plant-parasitic nematode biology conducted by the United States Department of Agriculture-Agricultural Research Service. Pest Management Science 59, 748–753 12846325 10.1002/ps.684

[CIT0015] Cortese MR Di Vito M De Giorgi C 2006 The expression of the homologue of the *Caenorhabditis elegans lin-45* raf is regulated in the motile stages of the plant parasitic nematode *Meloidogyne artiellia* . Molecular and Biochemical Parasitology 149, 38–47 16737746 10.1016/j.molbiopara.2006.04.003

[CIT0016] Curtis RHC 2008 Plant–nematode interactions: environmental signals detected by the nematode’s chemosensory organs control changes in the surface cuticle and behavior. Parasite 15, 310–316 18814700 10.1051/parasite/2008153310

[CIT0017] Dalzell JJ McMaater S Johnston MJ Kerr R Fleming CC Maule AG 2009 Non-nematode-derived double-stranded RNAs induce profound phenotypic changes in *Meloidogyne incognita* and *Globodera pallida* infective juveniles. International Journal for Parasitology 39, 1503–1516 19482028 10.1016/j.ijpara.2009.05.006

[CIT0018] Dalzell JJ Warnock ND Stevenson MA Mousley A Fleming CC Maule AG 2010 Short interfering RNA-mediated knockdown of drosha and pasha in undifferentiated *Meloidogyne incognita* eggs leads to irregular growth and embryonic lethality. International Journal for Parasitology 40, 1303–1310 20398669 10.1016/j.ijpara.2010.03.010

[CIT0019] Degenhardt J Hiltpold I Köllner TG Frey M Gierl A Gershenzon J Hibbard BE Ellersieck MR Turlings CJ 2009 Restoring a maize root signal that attracts insect-killing nematodes to control a major pest. Proceedings of the National Academy of Sciences, USA 106, 13213–13218 10.1073/pnas.0906365106 PMC2726344 19666594

[CIT0020] Dong L Huang C Huang L Li X Zuo Y 2012 Screening plants resistant against *Meloidogyne incognita* and integrated management of plant resources for nematode control. Crop Protection 33, 34–39

[CIT0021] Hannon GJ 2002 RNA interference. Nature 418, 244–251 12110901 10.1038/418244a

[CIT0022] Holden-Dye L Walker RJ 2011 Neurobiology of plant parasitic nematodes. Invertebrate Neuroscience 11, 9–19 21538093 10.1007/s10158-011-0117-2

[CIT0023] Hooper DJ 1984 Extraction of nematodes from plant material. In: Southey JF, ed.Laboratory methods for work with plants and soil nematodes. London: Ministry of Agriculture, Fisheries and Food–HMSO, 59–80

[CIT0024] Hooks CRR Wang KH Ploeg A McSorley R 2010 Using marigold (*Tagetes* spp.) as a cover crop to protect crops from plant-parasitic nematodes. Applied Soil Ecology 46, 307–320

[CIT0025] Horiuchi J Prithiviraj B Bais HP Kimball BA Vivanco JM 2005 Soil nematodes mediate positive interactions between legume plants and rhizobium bacteria. Planta 222, 848–857 16025342 10.1007/s00425-005-0025-y

[CIT0026] Huang GZ Allen R Davis EL Baum TJ Hussey RS 2006 Engineering broad root-knot resistance in transgenic plants by RNAi silencing of a conserved and essential root-knot nematode parasitism gene. Proceedings of the National Academy of Sciences, USA 103, 14302–14306 10.1073/pnas.0604698103 PMC1570184 16985000

[CIT0027] Johnston MJG Mcveigh P McMaster S Fleming CC Maule AG 2010 FMRFamide-like peptides in root knot nematodes and their potential role in nematode physiology. Journal of Helminthology 84, 253–265 19843350 10.1017/S0022149X09990630

[CIT0028] Kimber MJ McKinney S McMaster S Day TA Fleming CC Maule AG 2007 *flp* gene disruption in a parasitic nematode reveals motor dysfunction and unusual neuronal sensitivity to RNA interference. FASEB Journal 21, 1233–1243 17200420 10.1096/fj.06-7343com

[CIT0029] Li C Kim K Nelson LS 1999 FMRFamide-related neuropeptide gene family in *Caenorhabditis elegans* . Brain Research 848, 26–34 10612695 10.1016/s0006-8993(99)01972-1

[CIT0030] Lilley CJ Wang D Atkinson HJ Urwin PE 2011 Effective delivery of a nematode-repellent peptide using a root-cap-specific promoter. Plant Biotechnology Journal 9, 151–161 20602721 10.1111/j.1467-7652.2010.00542.x

[CIT0031] Luo M Dang P Bausher MG Holbrook CC Lee RD Lynch RE Guo BZ 2005 Identification of transcripts involved in resistance responses to leaf spot disease caused by *Cercosporidium personatum* in peanut (*Arachis hypogaea*). Phytopathology 95, 381–387 18943040 10.1094/PHYTO-95-0381

[CIT0032] Macosko EZ Pokala N Feinberg EH Chalasani SH Butcher RA Clardy J Bargmann CI 2009 A hub-and-spoke circuit drives pheromone attraction and social behaviour in *C*. *elegans* . Nature 458, 1171–1175 19349961 10.1038/nature07886 PMC2760495

[CIT0033] McVeigh P Geary TG Marks NJ Maule AG 2006 The FLP-side of nematodes. Trends in Parasitology 22, 385–396 16824799 10.1016/j.pt.2006.06.010

[CIT0034] McVeigh P Leech S Mair GR Marks NJ Geary TG Maule AG 2005 Analysis of FMRFamide-like peptide (FLP) diversity in phylum nematode. International Journal for Parasitology 35, 1043–1060 16076468 10.1016/j.ijpara.2005.05.010

[CIT0035] O’Halloran DM Burnell AM 2003 An investigation of chemotaxis in the insect parasitic nematode *Heterorhabditis bacteriophora* . Parasitology 127, 375–385 14636024 10.1017/s0031182003003688

[CIT0036] Pramanik MHR Nagai M Asao T Matsui Y 2000 Effects of temperature and photoperiod on phytotoxic root exudates of cucumber (*Cucumis sativus*) in hydroponic culture. Journal of Chemical Ecology 26, 1953–1967

[CIT0037] Rogers C Reale V Kim K Chatwin H Li C Evans P de Bono M 2003 Inhibition of *Caenorhabditis elegans* social feeding by FMRFamide-related peptide activation of NPR-1. Nature Neuroscience 6, 1178–1185 10.1038/nn1140 14555955

[CIT0038] Rosso MN Dubrana MP Cimbolini N Jaubert S Abad P 2005 Application of RNA interference to root-knot nematode genes encoding esophageal gland proteins. Molecular Plant-Microbe Interactions 18, 615–620 16042006 10.1094/MPMI-18-0615

[CIT0039] Tang CS Young CC 1982 Collection and identification of allelopathic compounds from the undisturbed root system of Bigalta Limpograss (*Hemarthria altissima*). Plant Physiology 69, 155–160 16662150 10.1104/pp.69.1.155 PMC426166

[CIT0040] Tian YQ Zhang XY Liu J Gao LH 2011 Effects of summer cover crop and residue management on cucumber growth in intensive Chinese production system: soil nutrients, microbial properties and nematodes. Plant and Soil 339, 299–315

[CIT0041] Troemel ER Kimmel BE Bargmann CI 1997 Reprogramming chemotaxis responses: sensory neurons define olfactory preferences in *C*. *elegans*. Cell 91, 161–169 9346234 10.1016/s0092-8674(00)80399-2

[CIT0042] Tsay TT Wu ST Lin YY 2004 Evaluation of Asteraceae plants for control of *M*. *incognita* . Journal of Nematology 36, 36–41 19262785 PMC2620738

[CIT0043] van de Putten WH Cook R Costa S 2006 Nematode interaction in nature: models for sustainable control of nematode pests of crop plants? Advances in Agronomy 89, 228–260

[CIT0044] Vicre M Santaella C Blanchet S Gateau A Driouich A 2005 Root border-like cells of *Arabidopsis*. Microscopical characterization and role in the interaction with rhizobacteria. Plant Physiology 138, 998–1008 15908608 10.1104/pp.104.051813 PMC1150414

[CIT0045] Wang KH Hooks CR Ploeg A 2007 Protecting crops from nematode pests: using marigold as an alternative to chemical nematicides. Plant Disease 35, 1–6

[CIT0046] Ward S 1973 Chemotaxis by the nematode *Caenorhabditis elegans*: identification of attractants and analysis of the response by use of mutants. Proceedings of the National Academy of Sciences, USA 70, 817–821 10.1073/pnas.70.3.817 PMC433366 4351805

[CIT0047] Yoshida K Hirotsu T Taqawa T Oda S Wakabayashi T Lino Y Ishihara T 2012 Odour concentration-dependent olfactory preference change in *C*. *elegans* . Nature Communications 3, 739 10.1038/ncomms1750 22415830

