# Supplementary material for: Lauric acid in crown daisy root exudate potently regulates root-knot nematode chemotaxis and disrupts Mi-flp-18 expression to block infection
Source: J Exp Bot. 2013 Oct 29;65(1):131–41. doi: 10.1093/jxb/ert356 (PMC3883285; doi:10.1093/jxb/ert356)
Supplement: Supplementary Data [file supp_65_1_131__index.html]

Lauric acid in crown daisy root exudate potently regulates root-knot nematode chemotaxis and disrupts Mi-flp-18 expression to block infection — Lauric acid in crown daisy root exudate potently regulates root-knot nematode chemotaxis and disrupts Mi-flp-18 expression to block infection — Supplementary Data 

# Lauric acid in crown daisy root exudate potently regulates root-knot nematode chemotaxis and disrupts *Mi-flp-18* expression to block infection

## Supplementary Data

Data files

**Files in this Data Supplement:**

- Supplementary Data - Supplementary Data
