# Supplementary material for: Lauric acid in crown daisy root exudate potently regulates root-knot nematode chemotaxis and disrupts Mi-flp-18 expression to block infection
Source: J Exp Bot. 2013 Oct 29;65(1):131–41. doi: 10.1093/jxb/ert356 (PMC3883285; doi:10.1093/jxb/ert356)
Supplement: Supplementary Data [file supp_ert356_jexbot104265_file001.pdf]

Journal of Experimental Botany

Lauric Acid in Crown Daisy Root Exudate Potently Regulates Root-knot Nematode Chemotaxis and Disrupts *Mi-flp-18* Expression to Block Infection

Linlin Dong, Xiaolin Li, Li Huang, Ying Gao, Lina Zhong, Yuanyuan Zheng, Yuanmei Zuo*

Table S1 The primers used in this study

Genes	Accession Number	Primers	Sequences(5'-3')
Mi-flp-18	**AY729022**	***flp-18*-F**	**5'-CAATTTTGGAGGACCTAGTG-3'**
		***flp-18*-R**	**5'-CAATGGCGGAAAAGACGATAG-3'**
		***T7- flp-18*-F**	**5'-TAATACGACTCACTATAGGGCAATTTTGGAGGACCTAGTG-3'**
		***T7-flp-18*-R**	**5'-TAATACGACTCACTATAGGGCAATGGCGGAAAAGACGATAG-3'**
		Re-flp-18-F	**5'-CCCAAGTTTGAGGGATATT-3′**
		Re-flp-18-R	**5'-ATTATTATGACCCGCCTCT-3′**
Actin	**BE225475**	***Re-actin-F***	**5'-GTTATTCTTTCACCGCAACCG-3'**
		Re-actin-R	**5'-GAATACCAGCAGATTCCATCCC-3'**
gfp	**AF234298**	***gfp*-F**	**5'-AGCGGCACGACTTCTTCA-3'**
		***gfp*-R**	**5'-GTGTGGACAGGTAATGGTTGT-3'**
		***T7-gfp*-F**	**5'-TAATACGACTCACTATAGGG AGCGGCACGACTTCTTCA-3'**
		***T7-gfp*-R**	**5'-TAATACGACTCACTATAGGGGTGTGGACAGGTAATGGTTGT-3'**
Mi-flp-1	**AY729023**	***Re-flp-1-F***	**5'-TTTGGGTGCTACAAGTGC-3'**
		Re-flp-1-R	**5'-ATTCCTTCTTCGTTTGAGGC-3'**
Mi-flp-7	**AY856132**	***Re-flp-7-F***	**5'-CACGCTTCTTCCTCTTCT-3'**
		Re-flp-7-R	**5'-GGTGCTCGTTTAGCCATA-3'**
Mi-flp-12	**AY804187**	***Re-flp-12-F***	**5'-CGTTTTGCTAGAGAATTTCCCG-3'**
		Re-flp-12-R	**5'-GAAGCATTTCCATTGCTCCC-3'**
Mi-flp-14	**AY907829**	***Re-flp-14-F***	**5'-GGCCGGGGGAGATGAAGA-3'**
		Re-flp-14-R	**5'-GCTTTCTCTTTTCCCGTC-3'**
Mi-flp-16	**EU549831**	***Re-flp-16-F***	**5'-GCAACAATCACAACAGCAAC-3'**
		Re-flp-16-R	**5'-GTTTGTGCTCTCTTTCCG-3'**

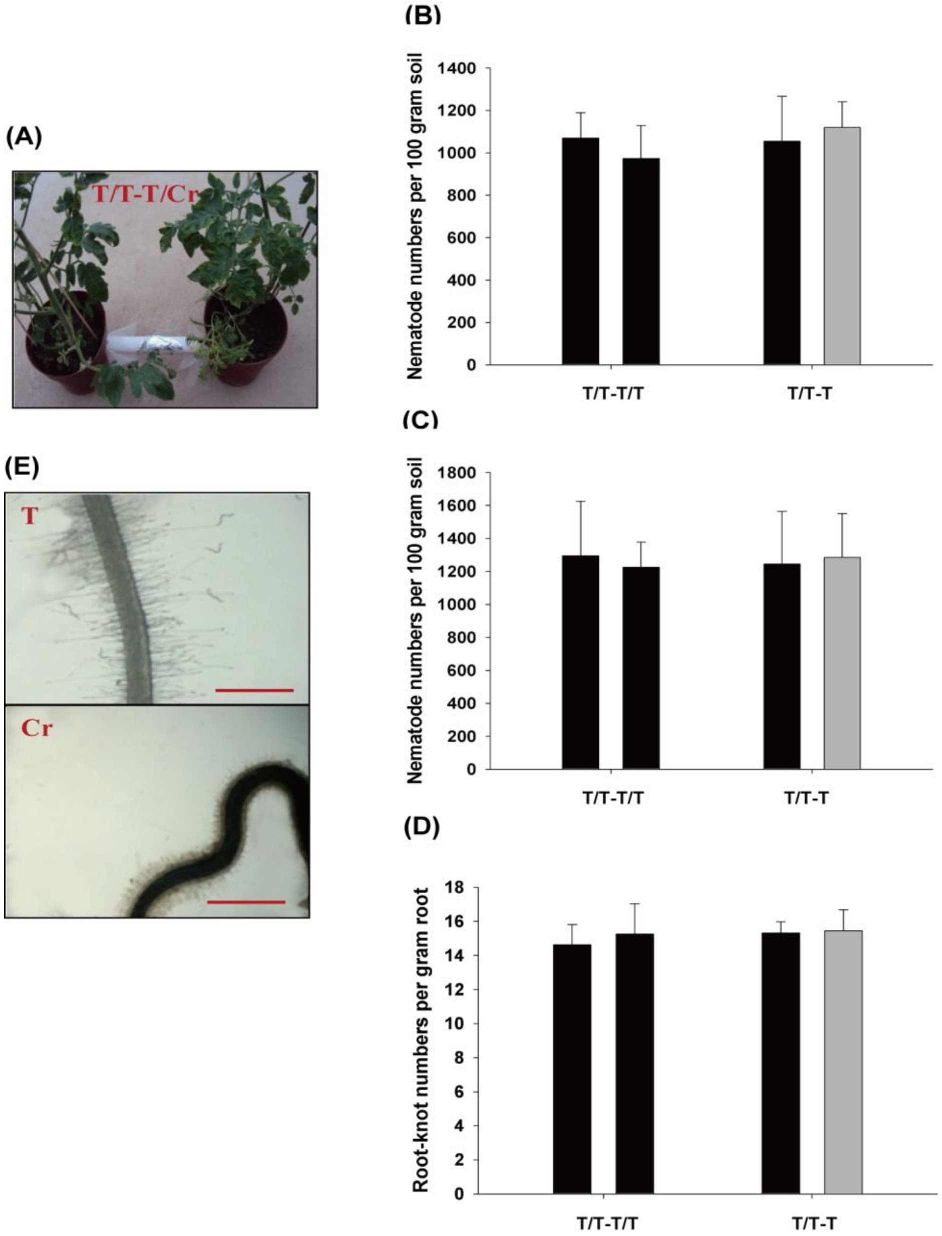

Figure S1. Root exudate plays important roles in blocking *M. incognita* infection. T: tomato; Cr: crown daisy; T-T/Cr: two tomato plants in the left pot and one tomato plant with five crown daisy plants in the right pot; T/T-T/T: two tomato plants in the left pot and two tomato plants in the right pot; T/T-T: two tomato plants in the left pot and one tomato plant in the right pot; (A) Depiction of the pot experiment linked by a tube. (B, C and D) Root exudate in the T/T-T/T and T/T-T combinations did not significantly affect the number of nematodes or root knots. (E) Root exudate of tomato and crown daisy regulated J2s chemotaxis in a Petri dish (scale bar, 1 mm). The value of each bar represents the mean ± SE of *n*=4, where a star denotes a significant difference at *P*<0.05.

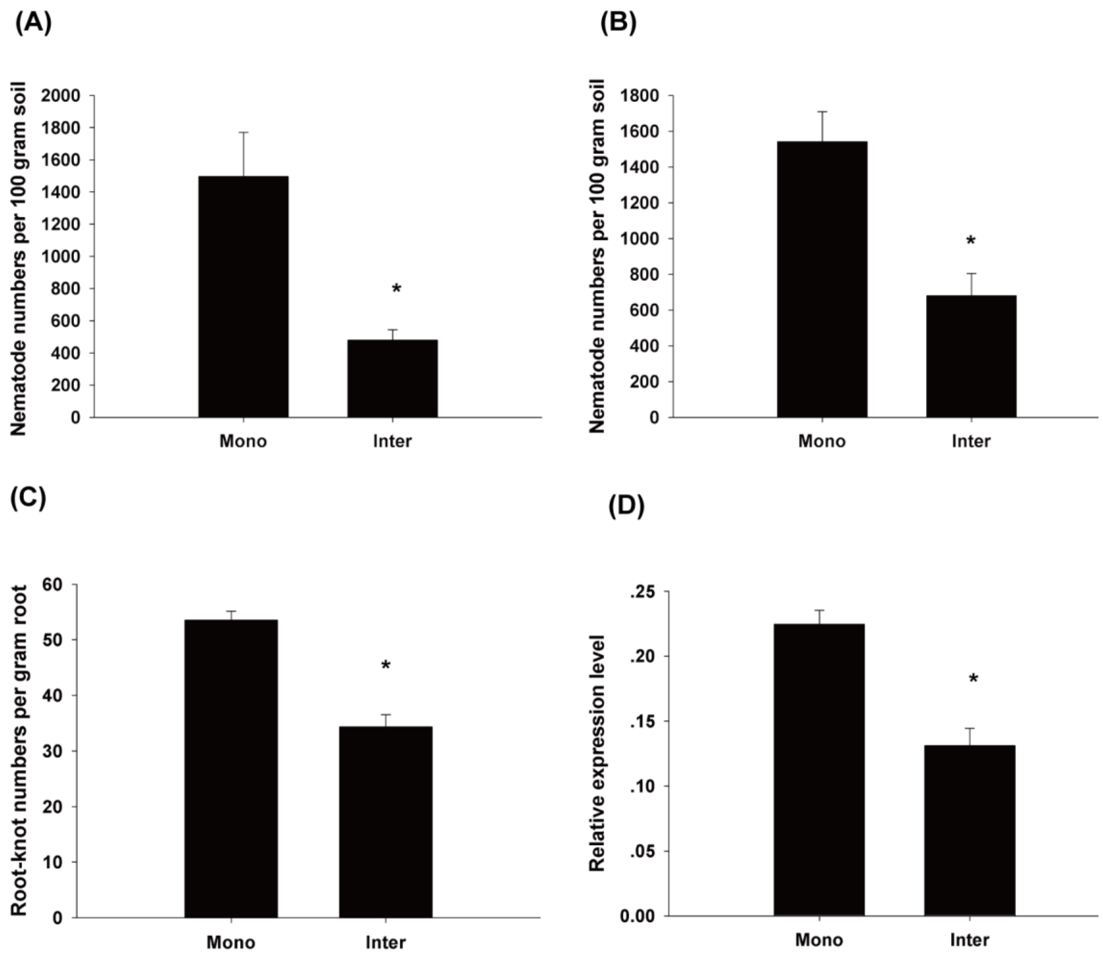

Figure S2. Root exudate in the tomato/crown daisy intercropping system reduced the number of nematodes and decreased the damage caused by nematodes by down-regulating *Mi-flp-18* expression in the pot experiment linked by a tube during 2009. Mono: monocropping. Inter: intercropping. (A, B) Root exudate reduced the numbers of nematodes in the tube soil and pot soil. (C) Root exudate reduced the number of root knots. (D) *Mi-flp-18* expression during parasitism.

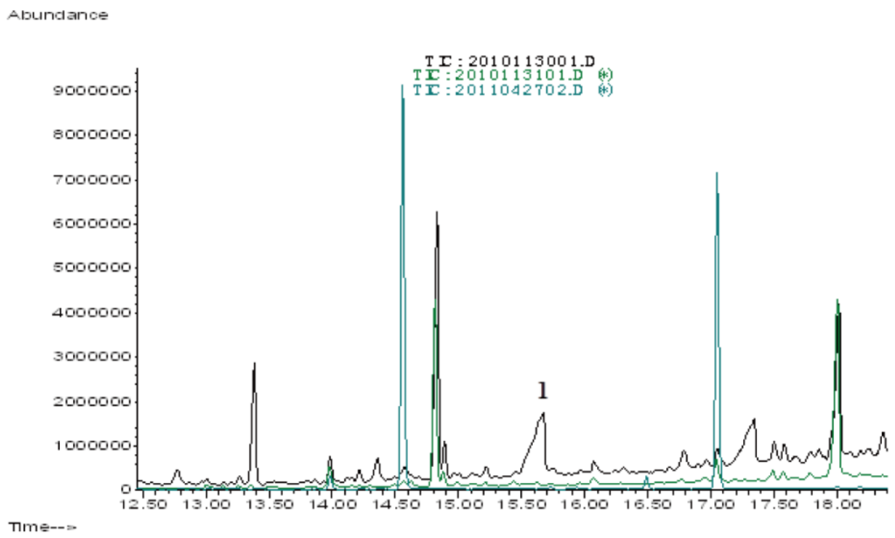

Figure S3. Gas chromatogram of root exudate from tomato and crown daisy by GC-MS assay. The numbers 001, 101, and 702 represent the gas chromatograms of crown daisy, tomato and the control, respectively. 1: lauric acid.

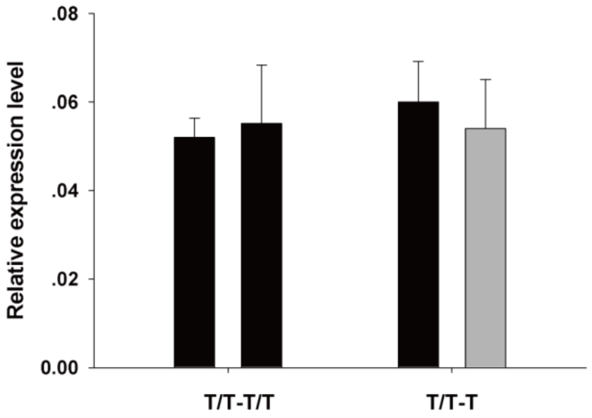

Figure S4. *Mi-flp-18* expression is downregulated during parasitism in the tomato/crown daisy intercropping system. T: tomato. T/T-T/T: two tomato plants *versus* two tomato plants in the pot experiment linked by a tube. T/T-T: two tomato plants *versus* one tomato plant in the pot experiment. The value of each bar represents the mean ± SE of n=4, where stars denote a significant difference at P<0.05.
